# Direct 16S rRNA-seq from bacterial communities: a PCR-independent approach to simultaneously assess microbial diversity and functional activity potential of each taxon

**DOI:** 10.1038/srep32165

**Published:** 2016-08-31

**Authors:** Riccardo Rosselli, Ottavia Romoli, Nicola Vitulo, Alessandro Vezzi, Stefano Campanaro, Fabio de Pascale, Riccardo Schiavon, Maurizio Tiarca, Fabio Poletto, Giuseppe Concheri, Giorgio Valle, Andrea Squartini

**Affiliations:** 1 Department of Biology, University of Padova, Padova, Italy; 2 Department of Biotechnology, University of Verona, Verona 37134, Italy; 3 RWL Water/Eurotec WTT, Water Treatment Technologies, S.r.l., Padova, Italy; 4 Department of Agronomy Animals, Food, Natural Resources and Environment, DAFNAE, University of Padova, Legnaro (Padova) Italy

## Abstract

The analysis of environmental microbial communities has largely relied on a PCR-dependent amplification of genes entailing species identity as 16S rRNA. This approach is susceptible to biases depending on the level of primer matching in different species. Moreover, possible yet-to-discover taxa whose rRNA could differ enough from known ones would not be revealed. DNA-based methods moreover do not provide information on the actual physiological relevance of each taxon within an environment and are affected by the variable number of rRNA operons in different genomes. To overcome these drawbacks we propose an approach of direct sequencing of 16S ribosomal RNA without any primer- or PCR-dependent step. The method was tested on a microbial community developing in an anammox bioreactor sampled at different time-points. A conventional PCR-based amplicon pyrosequencing was run in parallel. The community resulting from direct rRNA sequencing was highly consistent with the known biochemical processes operative in the reactor. As direct rRNA-seq is based not only on taxon abundance but also on physiological activity, no comparison between its results and those from PCR-based approaches can be applied. The novel principle is in this respect proposed not as an alternative but rather as a complementary methodology in microbial community studies.

Since its advent during the 1970s, 16S-rRNA sequencing has represented a fundamental step for bacteria identification and essential information for their classification^1^,^2^. The structure of the 16S-rRNA genes is defined by an alternation of highly-conserved and hypervariable regions, reflecting the effects determined by function-related constraints in the former and those allowed by their absence in the latter^3^,^4^,^5^,^6^. The estimated substitution rate is ~7000 times higher for the hypervariable regions than the highly-conserved ones^7^,^8^ and these genetic differences have been considered to reflect, for most bacteria, genome divergence^9^, making the 16S sequence an ideal proxy to achieve a trustworthy level of taxonomic information. Widely recognized as the ‘gold standard’ for bacterial identification due to these specific features, 16S rRNA gene sequencing has been the target of countless studies in which universal PCR primers have been applied and the resulting partial 16S gene amplicons, encompassing hypervariable regions, used to infer taxonomic identifications based upon bioinformatics alignments against sequence databases^10^,^11^,^12^,^13^.

With the development and improvement of sequencing platforms the nucleotide sequencing throughput has today reached millions of reads per single run. Investigation strategies have likewise been adapted to configure universal primers and PCR-products to fit the different platforms’ technical features. Primers aiming at offering an as wide as possible coverage in the NGS era have been reported^14^. Effects of DNA extraction methods have also been examined^15^. The increasing amount of data determined the development of several bioinformatics pipelines. Frequently updated online databases and tools such as CAMERA^16^, The RDP-Ribosomal Database Project^17^, SILVA (http://www.arb-silva.de), MG-RAST^18^ have become fundamental for sequence collection and analysis, while informatics tools as the MOTHUR^19^ and QIIME^20^ suites, or the EMIRGE^21^ 16S assembler were developed in order to analyze single reads, assembled paired-end or assembled full-length 16S-rRNA genes. While new sequences are regularly added to databases (more than ten million 16S-rRNA gene sequences are currently available, source: ncbi.nlm.nih.gov), a series of intrinsic limitations affecting the different experimental protocols have been highlighted^22^,^23^,^24^,^25^,^26^,^27^,^28^. These aspects prompted both protocol improvements^29^ and suggestions for denoising or data mining^30^,^31^,^32^,^33^.

A main drawback of the existing methodologies, which are all based on PCR, is the *a priori* knowledge assumption required for universal primers design. Yet the result of every PCR-dependent approach is inherently affected by the fact that the ‘universality’ of any primer pair is not absolute. As a consequence, the current experimental protocols may determine an altered or incomplete estimation of the true biodiversity of a given environmental sample^34^. Indeed, an uneven primer annealing performance during the first PCR cycles can severely bias the final community members proportions^35^. Moreover, as primer design is based on existing knowledge, such tools remain self-referenced and do not guarantee the discovery of species whose sequence could diverge from established consensus. Essentially, current approaches to assess environmental bacterial species presence and abundance are undermined by a common principle that could affect the conclusions drawn on community structure in terms of taxa richness and evenness.

While PCR-amplified 16S-rRNA gene sequencing can be useful for straightforward classification of purified isolates, accurate taxa quantification in environmental samples may be also affected by the possible occurrence of multiple identical^36^,^37^ or different^38^ copies of ribosomal operons^39^,^40^,^41^. A deeper level of information was obtained comparing 16S-rRNA and cloned 16S-rRNA genes, but was still dependent on a PCR process, as total RNA was retrotranscribed and the 16S specific cDNAs were then amplified by universal primers^42^,^43^. An alternative way to assess bacterial species identity is to rely on metagenomic surveys^44^,^45^ in which however bacterial 16S genes are overly diluted into the genomic DNA. Besides, such approach requires high numbers of reads, deep computational capacity and high prices^46^. Also in metatranscriptomics the importance of including rRNA sequencing data along the messenger transcript analysis has been advocated but data have not been compared at quantitative level^47^. Rather than relying on the uncertainties of primer matching on the rDNA, a more trustworthy assessment of the bacterial populations can be sought in the direct sequencing of its rRNA product. An added value of this principle relies on the hypothesis that each taxon presence and its relative activity within a community should be proportional to the number of its ribosomes. According to several evidences the capacity to provide a quick response to the environmental changes reflects the bacterial whole genome and transcriptome organization^48^. As fundamental molecules, the transcription rate of rRNAs is subjected to a direct modulation that promptly reacts to any change which could affect cell survival^49^. However the link between rRNA content and actual microbial activity has been challenged, pointing out that the presence of rRNA should be regarded as indicative of protein synthesis potential rather than of realized protein synthesis^50^.

Relying on the tenet that the most active bacteria within any community, or, more adequately, those with the highest potential for protein synthesis, would have correspondingly higher rates of transcribed 16S-rRNA, we propose the use of such PCR-independent methodology to perform unbiased environmental diversity assessments via direct rRNA sequencing and to quantify each taxon using Next Generation Sequencing high throughput platforms. The advantages of the methodology are firstly that the approach is totally independent of PCR and consequent preferential primer matching issues towards given species. This makes the principle also able to detect any rRNA regardless of its evolutionary conservation and/or similarity to any previously known consensus sequence. Secondly, the method assesses the quantitative occurrences of taxa on the basis of their ribosomes abundance. This solution, which also solves the issue of a variable number of ribosomal operons across species, provides a view of the functional potential of each species activity within the community. We tested the method on mixed bacterial populations developing within an anammox (*an*aerobic *amm*onia *ox*idation) industrial-scale bioreactor sampled at two different time-points. The rRNA-seq libraries were sequenced with an AB SOLiD 5500 XL platform.

In order to visualize also the data that would result from a corresponding standard PCR-dependent NGS pyrosequencing approach, in parallel we used universal primers to build amplicon libraries that were sequenced in a 454 FLX system. The choice of SOLiD for the rRNA-seq was favoured by its ultra-high throughput, assuring a thorough and sensitive detection of the widest array of metabolically-involved bacteria, in case the massive amount of 16-rRNAs produced by the most active cells would mask the less active ones. Nonetheless the protocol is equally suited to perform in any other sequencing platform, as further shown by our virtual in-silico simulations for the method validation. As regards the use of SOLiD, in literature it has also been shown to be validly applied in a classic amplicons approach, yielding results comparable to the 454 FLX system^51^. The choice of a confined environmental system with a well-characterized biochemical stoichiometry also allowed each method’s results to be verified under known technological performances. Since the analysis was coupled with the operative cycle of an actual industrial anammox bioreactor, the two time points (day 154 and 189) were selected upon the daily screening of the nitrogen abatement performance (ammonium, nitrite and N_2_ dynamics) and represent two stages (mid-point and early mature stage) of the process. This was intended to couple the sequencing data with the expected prevalence of an anammox-effective community from the physiological standpoint.

## Results

### Sequencing and alignment to references database

As mentioned, the approach was dual: a pyrosequencing with Roche titanium of the 16S amplicons and a PCR-independent rRNA direct sequencing. Regarding the PCR-based 454 reads, after trimming and filtering procedures, a total of 4,636 sequences were obtained for the first sampling time (day 154) and 3,397 sequences for the second (day 189). All sequences, including singletons, were kept in order to achieve an exhaustive representation of the raw data.

From the direct rRNA-seq protocol, after the cleaning procedure, a total of 43,259,527 (primer F3), 61,943,781 (primer F5) and 43,713.,403 (primer F3), 53,468,401 (primer F5) SOLiD reads were obtained for the first (day 154) and second (day 189) sampling times, respectively.

**Figure 1:**
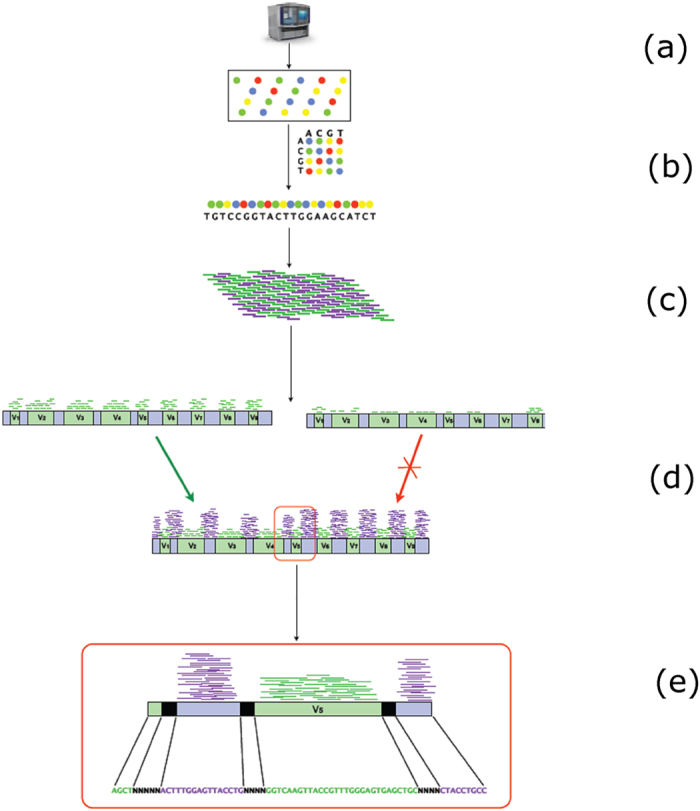
Outline of the rRNA sequencing and annotation procedure. Purple lines: not-uniquely aligned reads. Green lines: uniquely aligned reads. The latter, being specific for a single target within the database, fall on hypervariable regions (vs). (**a**) Paired-end SOLiD RNA-sequencing; (**b**) Double-encoding and read alignments on a 16S-rRNA reference database; (**c**) Selection of candidate subjects. Only subjects covered at least 10% of the total length by unique-aligned reads were selected for the second screening; (**d**) Selection of confirmed subjects. Not-uniquely-aligned reads information was used to confirm the subject. Sequences with at least 50% of total length covered were considered for the annotation; (**e**) Sequences annotation: subjects aligned over encoded and not-encoded (NNN) regions were annotated by CAMERA.

To maximize biodiversity representation and avoid loss of information due to the lack of some reference sequences in each of the specific databases, the 16S reference set against which the screening was performed was obtained by pooling the two DDBJ and RDP individual repositories. A cluster analysis was then performed considering 97.5, 95, 92, 90, 88% of similarity as thresholds to verify whether the different redundancy levels would affect the matching outcome. The procedure outline is shown in Fig. 1. The lowest number of uniquely-aligned reads was obtained for the 97.5% similarity threshold for each sample, while the highest was found for 90%. The classification resulting for the 95% level of sequence similarity, was the one who gave the highest number of taxonomically-identified database subjects and was therefore chosen as reference to assemble data. Details on the effect of the clustering threshold on the number of identified database subjects are given in Supplementary Note 1.

As regards the in silico validation of the primers efficiency and their comparison with their cognate published versions S-D-Bact-0341-b-S-17 and S-D-Bact-0785-a-A-21^52^, as well as with the widely used pairs UF89F-B1046R^53^,^54^ and 515f-806R^55^, the results of the ProbeMatch and TestPrime analyses confirmed a validly comparable performance. Results of these tests are available in a spreadsheet as Supplementary material dataset S2 in silico primers performance analysis.

### Amplicons and rRNA-seq sequence annotation results

**Figure 2:**
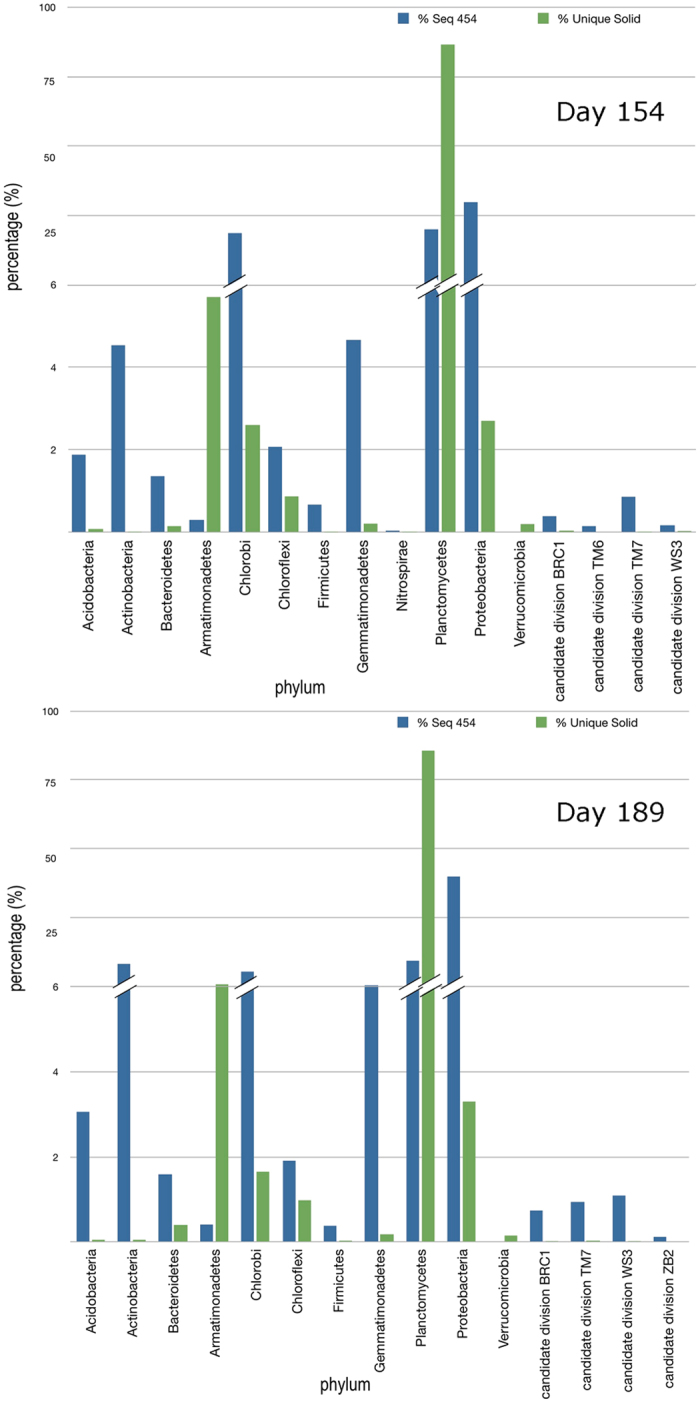
Percentages of taxa assigned through database alignment of sequences obtained by 454 (PCR-based) amplicon pyrosequencing (blue bars) and by SOLiD direct rRNA-seq (green bars) for the phyla of the bacteria superkingdom occurring in the anammox bioreactor sludge at the two sampling dates. Note the broken scale step to magnify the region below the 6% abundance threshold, separated from the top which shows the 100% value.

The taxonomic classification at phylum level of both amplicons and rRNA-seq data is shown in Fig. 2 and in Table 1. The full list of identified taxa is available in Supplementary material dataset S1, taxa lists, in which abundance percentage of each case and similarity levels to each corresponding database subject (phylum and genus rank levels) are shown.

The images show the results obtained by each method, whose outputs as mentioned are not meant for direct comparison since the rRNA-seq relies non only on cells abundance but also on their ribosome number. Essentially, while the PCR-based pyrosequencing assigned the dominant abundance to Proteobacteria, with 34.06% and 43.4% for sampling times day 154 and day 189, respectively, the scores of Proteobacteria in the activity-related direct rRNA-seq were more than ten-fold lower, resulting as 2.7% and 3.3%. In these the overwhelmingly dominant phylum was instead Planctomycetes, amounting to 87.36% and 86.44%, respectively. Interestingly, Armatimonadetes were represented by more than 5% of the rRNA-seq reads while appearing scarcer in the PCR-dependent analysis (<0.5%). Chlorobi displayed an inverse trend, scoring 23.6% and 10.9% by PCR-based pyrosequencing and only 2.6% and 1.65% by rRNA-seq. Some taxa were identified by just one of the approaches, e.g. Verrucomicrobia, detected only in the rRNA-seq data at both sampling times. Acidobacteria, Actinobacteria and Gemmatimonadetes appeared comparatively over-represented in 454 amplicons data with respect to rRNA-seq.

To quantify abundance of genera identified by rRNA sequences, only subjects covered by at least 200 SOLiD reads were considered, to discard low-coverage taxa. Using this threshold, 116 genera and 122 genera were identified for day 154 and day 189 samples, respectively.

This genus-level taxonomic classification (full list given in Supplementary dataset S1) highlighted the following; regarding overall diversity, for the amplicons, 42 genera represented by more than 10 sequences and 99 genera by more than 1 sequence were detected for the first time point while 39 and 92 were observed for the second.

**Figure 3:**
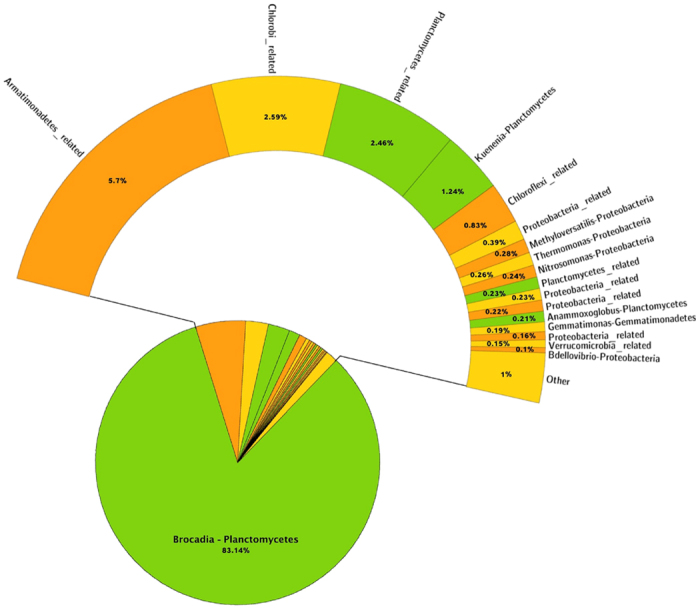
Percentages of taxa assigned at genus level by the rRNA-seq. Day 154 sampling.

**Figure 4:**
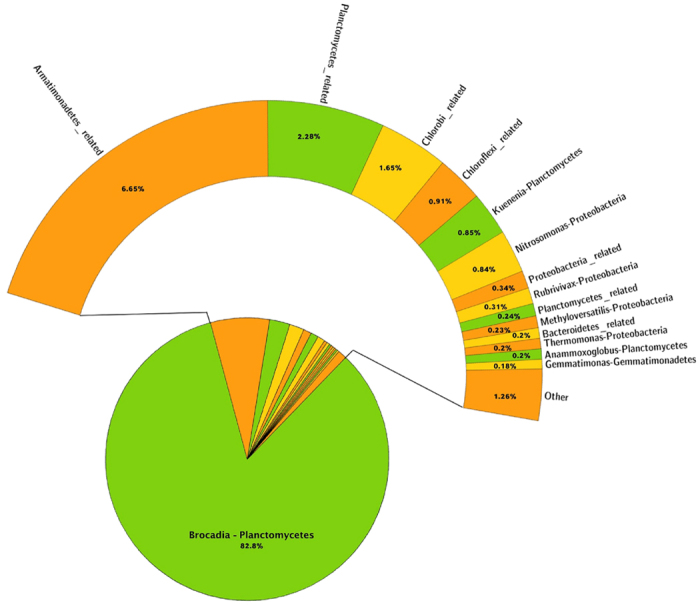
Percentages of taxa assigned at genus level by the rRNA-seq. Day 189 sampling.

In the PCR-based pyrosequencing, the genera of Proteobacteria (score leaders at phylum level), resulted as being mostly represented by *Methyloversatilis* sp. (14.54% at day 154 and 11.60% at day 189). Remaining Proteobacteria included unidentified genera within Rhodocyclales and Rhodobacterales orders and several others with abundances from 2.67% downwards. The dominant member of Planctomycetales in the 454 sequencing was a ‘*Candidatus* Brocadia’ with a decreasing trend from day 154 (24%) to day 189 (14%). The most abundant genera for the rRNA-seq data are shown in Figs 3 and 4. In these cases the dominant Planctomycetes was *Candidatus* Brocadia and the decreasing trend observed in the amplicon-based sequencing was not present between the two time-points since this genus was equally represented in both samples (around 83%).

Moreover, rRNA-seq identified other taxa belonging to Planctomycetales that were not found using the amplicons method; a member of the Kueneniaceae family and a *Candidatus* Kuenenia were identified at both sampling times. This is in part also in line with the higher sampling depth inherent to the higher throughput of the SOLiD approach. The full list presents other cases of Planctomycetes (below 0.2% but still represented by several thousands of unique aligned reads) including other *Candidati* such as Anammoxoglobus, Scalindua and Jettenia, and a member of the Planctomycetes class Phycispherae.

It is worth noting the presence of a Chlorobi-related organism whose match in sequence similarity with database subjects was not higher than 92% and the annotation did not reach levels deeper than the phylum rank. It could therefore represent another as yet uncharacterized organism enriched in this anammox bioreactor. This taxon scored 30% of the bacterial population in the 454 amplicon sequencing, both methods confirming its consistent presence.

The Armatimonadetes-related bacteria, which are the second most abundant identified organisms in the direct rRNA-seq analysis, show the opposite trend (under-represented in the amplicon-based sequencing).

As for Chlorobi, this taxon’s homology to known records arose only at phylum level and qualified it as another important anammox process-related novel bacterium as well as another case (besides Planctomycetes) for which proportional detection in PCR-based studies might have suffered a certain bias.

The results also included a considerable number of rRNA reads that did not align with the databases. For the sampling time at day 159 these were 65,711,669 out of a total of 105,203,308 (62.4%) and for day 189 there were 73,694,386 out of a total of 97,181,804 (75.8%). In part this is expected to be a consequence of the alignment/annotation procedure required downstream this specific NGS output, since the SOLiD principle of ligation and two-base color coding generates 70 + 30 bases reads that require an indirect assembling targeting the unique alignments.

The method accuracy in sequence annotation was in parallel satisfactorily validated by a series of in-silico simulation tests whose rationale and results are shown in Supplementary information (Supplementary Note 2).

## Discussion

It needs to be first reminded that the two methods applied here have a substantial difference; the DNA-targeting analysis scores abundance as essentially linked to rRNA gene numbers, which are influenced by ribosomal operon copy number in the genome, and cell number. Conversely the quantitative data produced by the rRNA-seq are centered on potential protein synthesis activity as a function of the number of ribosomes per single cell and are not necessarily coupled to total cell number nor population growth. Therefore a net comparison of the two strategies can not be performed since the two methods draw their data from independent principles. Consequently while we discuss the outcome of each method, even though we point out at ‘differences’, no measure of the PCR bias can be inferred from those data. The amplicon pyrosequencing experiment was in fact included as a referential example of the classic standard procedure that has been used in the past decade for a majority of environmental microbial analyses. As a consequence, besides the fact that any differences in the measured community composition could be due to ribosomal activity and/or due to effects of PCR bias, the use of different sequencing technologies and downstream bioinformatics processing pipelines can also exert an effect in the final annotated outcome. Therefore the PCR-mediated analysis must not be mistaken for the ‘control’ for the rRNA-seq. The data ought to be regarded as those from two different, independent, possibly complementary strategies.

Keeping in mind these premises and restraining from any directly comparative evaluation, we indeed observe that, as expected, the two sequencing strategies, amplicons vs transcribed 16S-rRNA, yielded marked differences in terms of community taxonomic proportions and deducible bacterial physiology in the environment of choice. Being this an anammox bioreactor constantly monitored for nitrogen feeding and abatement, it offered the advantage of knowing *a priori* which main microbial activities were expected and, consequently, which microbial guilds could be, at least in part, expected to represent the assemblage.

While amplicon-based sequencing reported a predominant population of Proteobacteria outnumbering the sequences from the anammox *Candidatus* Brocadia, direct rRNA-seq instead indicated the overwhelming majority of reads as belonging to Planctomycetes, i.e. the phylum known to encompass all presently known anammox-effective bacteria. Such proportions were confirmed at both sampling times and are backed up by the large number of sequences obtained, since both methods exploit next generation sequencing technologies. The premises thus appeared correct: the newly proposed approach directly targeting ribosomal transcription rate as simultaneous representation of bacterial presence *and* dynamic potential for physiological activity, proved suitable to the scope as the main expected metabolism in the chosen environment was indeed that of the Planctomycetes. At the same time, as the method is independent of any primer annealing strength issues and PCR fidelity, it avoids all consequent biases related to amplification-based analyses that are currently still the standard in studies of this kind.

Besides the differences in phyla proportions, the SOLiD sequencing approach and the associated bioinformatics pipeline also showed higher resolution in extracting the diversity of this system. Indeed, while from amplicon pyrosequencing a single type of Planctomycetales could be individuated as representative of the phylum, the rRNA-seq reads revealed that two other anammox-related genera were present and actively maintained a high protein synthesis potential in this environment. The higher diversity observed is in any event also in line with the higher throughput of the system in itself, providing a deeper detection of the rare taxa.

Table 1 allows to observe the results of the two approaches. The method is indeed introducing the physiological aspect of taxon activity within the community besides that of presence assessment. As mentioned, inevitably this prevents the possibility of a net comparison of the two methods, and any consideration in this respect needs to be kept in mind when following these data. Planctomycetes and Armatimonadetes, which were the most abundant from the RNA sequencing at both sampling times, are apparently highly metabolically active groups. It is noteworthy that these phyla feature DNA/rRNA-based results ratios <1, i.e. their abundances obtained by rRNA-seq were higher than those generated by PCR-based DNA sequencing. Moreover an important consideration concerning Armatimonadetes is that those are the group that, among all taxa identidfied in this bioreactor, displayed the most severe predictable matching inefficiency from the *in silico* primer analyses. Data are shown in Supplementary material dataset S2, where scores as low as 34% (Silva) and 19% (RDP) databases were put in evidence. This supports the value of the present method as suited to uncover and reveal the importance of bias-sensitive phyla in community analyses.

The other phyla displayed PCR-dependent frequencies higher than those yielded by direct rRNA-seq, and their ratios were not constant, spanning from extremely high disproportions e.g. Actinobacteria, followed by Firmicutes, Acidobacteria, Bacteroidetes, Proteobacteria, to lower but appreciable differences.

Besides the dominant Planctomycetes, Chlorobi-related bacteria were highly detected with the direct RNA-seq. This group has been reported as presumably related to granules formation^56^ and these structures are thought to have a fundamental role for a high anammox reaction rate^57^. *Methyloversatilis* presence could instead be related to concurrent denitrification processes^58^. The consumption of carbon sources^59^ may characterize a “denitrifiers-shift” to other species such as Actinobacteria-Solirubrobacterales as described for other substrate-specific communities^60^. An increase of these taxa is noticeable from the first to the second sampling time point, although further replication effort would be necessary to prove the significancy of such apparent trends. Changes within anammox-related bacteria, also depending on different substrate affinity has been described^56^,^61^. In the present analysis the dominant taxa appear to maintain a relatively stable 16S-rRNA transciptional rate throughout the two sampling points, including also the Chlorobi phylum was confirmed by both sequencing approaches.

The sampled microenvironment was deemed an ideal setting for a hypothesis-testing approach as the biochemical data reflected an overtly active anammox metabolism. Besides corroborating Planctomycetes as main players and confirming Chlorobi as associated group as proposed in the literature, we detected a further relevant and supported occurrence of the phylum Armatimonadetes, not previously reported in anammox communities but apparently important as it was the second most active/ribosomally represented phylum after the Planctomycetes at both sampling times. This strengthens the suitability of the approach in estimating taxonomic groups that were rarely detected by techniques relying on PCR.

In examining the data stemming from the two approaches it should be considered that one of the drawbacks of the PCR-based extant methods is the fact that bacteria can have more than one copy of ribosomal genes operon and that this creates a further bias as it proportionally leads to an overestimation of cell counts for cases with multiple operons. With the rRNA-seq approach such issue is bypassed by targeting directly the transcribed rRNA molecules as a genuine function of that species contribution to potential physiological activity at that time within that environment. Planctomycetes are anyhow reported to possess a single copy of rRNA operon and their prominent position in the community is to be seen as related to the combination of high expression rate and high attained cell numbers. Considering that their generation time involves a doubling every 9–11 days, and that in the initial inoculum (sampling time day 1) they were at very low levels as judged by quantitative PCR using Planctomycetes–specific primers (data not shown), we can estimate that their population dynamics has unfolded steadily and that the rRNA sequencing at five and six months have efficiently revealed their outcome. In general terms and for phyla in which one could not have information on the number of ribosomal gene operons, it can be taken as a reference point that the number of rRNA copies in each bacterial cell is bound to be always substantially higher than the number of starting rDNA genes. In *E. coli* the average number of ribosomes is estimated as 18,700^62^ with wide fluctuations from 6,700 to 71,000 per cell depending on growth phase^63^, while the number of rRNA operon copies in bacterial chromosomes spans between 1 and 15^64^. PCR can multiply exponentially such rDNA with a performance relying upon the chances of primer matching strength. For rRNA-seq, proportions instead inherently reflect only two factors: a) taxon abundance and b) level of gene expression within the system, mirrored by the dynamically fluctuating number of ribosomes.

Essentially it is helpful to stress that in the PCR approach the number of sequences obtained for a given taxon obeys to the equation:


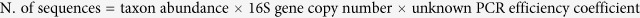


While in the RNA based approach it is:


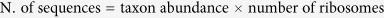


As concerns some technical considerations, the method was here coupled to a SOLiD platform, but the approach is amenable and compliant to any RNA-seq protocol and any type of high throughput Next Generation Sequencing platforms. Even if the color-space format does not allow a ribosomal RNA assembly, usage of a software like EMIRGE could be tested in a 16S-rRNA-seq Illumina run or in other base-space reads output. The following further details can be outlined; the native format of the ABI SoLID technology yields data in color space, which need to be translated to base space (nucleotides). The output of the method presented here (conversion of reads in a merged and workable sequence) is also suitable to be used with any of the available tools and utilities for sequence classification as it is in the form of a plain (base-encoded) gene sequence and is even longer than the average read produced by any current sequencing machine. In addition its abundance also entails a datum on gene expression within the sampled environment. The approach principle is such that it does not allow any chimera formation in the sequencing stage, thus avoiding another drawback of amplification-based methods.

The analysis run on databases clusterized at various degrees of identity stringency (Supplementary material Technical note 1) showed that, notwithstanding the decreasing size of the resulting numbers of subjects (from over 120,000 to less than 14,000) the number of identified sequences stayed practically constant, indicating a strong degree of independence of the protocol from the database size. Therefore any multi-FASTA assemblage of 16S references can be indicated as a valid substrate for the identification analysis as well as any online 16S amplicon annotation tool could be used.

In analyzing defined ribosomal RNA bands obtained upon electrophoretic separation, it should be also considered that the presence of bacteria containing self-splicing introns and protein coding regions within their rRNA could give rise to transient transcripts of unconventional sizes, but when these are mapped as metatranscriptomic sequences to assembled 16S rRNA genes it appears that those insertions are not retained in transcribed RNAs and are probably rapidly degraded^65^; their occurrence is therefore not affecting approaches as the one presented in our study.

Summarizing the evidences and issues emerging from the present analysis, rRNA gene amplification studies provide a deep level of information on the diversity of biological systems. Nevertheless, as underlined in many studies cited above, in relation to potential biases and limitations, data trustworthiness and results interpretation under current practices are still affected by considerable levels of uncertainty and complexity. The use of 16S-rRNA as a prokaryotic universal barcoding remains a reference asset but its use is affected by critical issues mostly consisting in primer matching biases due to the degeneracy of the conserved regions and to the consequent non-universality of any currently known primer pair. This in turn influences dramatically the first PCR cycles and can exponentially carry over an error in the end point proportions of each taxon with respect to its original values in the sampled community. Further critical issues concern chimera formations during PCR and sequencing. Another limit of any PCR-based census based on the mere gene presence is that no information is conveyed on the actual level of activity (or quiescence) of that given cell. This prevents to infer anything about its consequent contribution to its ecosystem physiology and ultimately to the ecology of the environment under study. Even in the presence of the most trustworthy data a further critical aspect, is the proper handling of the massive raw outputs of the sequencers and the correct probing of the available databases, to convert reads into exact and meaningfully annotated information. The method presented herewith addresses all these criticalities and considers alternative solutions to reduce their impact. The high throughput of NGS machines has been coupled with a transcriptomics-driven approach where taxonomical assignment is coupled to activity assessment. A purposely-elaborated reads-processing workflow is followed by a taxonomical assignment pipeline which exploits jointly two major sequence databases.

The high number of RNA sequences that did not align suggests considerations over the potentialities to further exploit this kind of approach. Besides accounting for a rate of sequencing errors, the reason for not matching ribosomal databases could be dual. Namely (a) belonging to taxa which have yet not been detected as their sequence diverges enough from known 16S primers as well as from extant database subjects under the alignment strategy and database clustering stringency used for annotation in the present rRNA sequencing approach; (b) belonging to a co-purified fraction of mRNA. However we deem this of minor extent for the following reasons: ribosomal RNA is the dominant and most stable form of RNA within cells (>95% of the total ribonucleic acids), it can amount from 12 to 48% of the bacterial biomass dry weight (ranging upon physiological activity). Moreover in stained RNA gels the two bands of 23S and 16S visually represent the strikingly dominant bands and are well resolved from each other as well as from the messenger which is mostly in a higher and rather faint smear pool. Besides, mRNAs, being destined to rapid turnover, are by nature more sensitive to degradation than their structural ribosomal counterparts.

## Conclusions

The direct rRNA-seq approach compared to amplification-based sequencing has shown unique results in the detected community composition, consistently with its simultaneous assessment of presences and physiological rate of metabolic activity potential of bacteria. Supposedly this result is also achieved by virtue of its independence from possible PCR-related biases for given taxa. Seeking to mention possible limitations of the method, one could comment that, being based on rRNA molecules, if bacteria were very quiescent their signal could be low; however this is also the strength of the method, i.e. it is targeting those species featuring high protein synthesis potential no matter their rarity. In this respect the DNA-based PCR-dependent methods remain valuable companion procedures and complementary approaches to extract information from inactive/dead cells or from preserved cell-free DNA.

As mentioned, the reliability of all taxonomy-assigning methods rests upon the availability of a large and precisely annotated database. A method that is not primer-dependent and aims straight at the rRNA also offers the possibility of uncovering novel lineages of bacteria previously overlooked by possible inadequacy of primers, all of which are inevitably designed upon existing knowledge that results in a ‘catch 22’ situation of self-referencing information. Overcoming these limits is one of the goals of microbial taxonomy. The high number of low-identity matching sequences and that of non-aligning reads found in the present analysis could in part be due to the peculiar bioreactor habitat, or to possible non –ribosomal contaminants or to limits in the reads annotation pipeline. Nevertheless dedicated direct approaches are envisaged as necessary to tap on the unknown reservoir of unculturable bacteria with un-amplifiable 16S rRNA genes that could inhabit environments that have hitherto been explored mainly with PCR-based methods.

## Materials and Methods

### Anammox bioreactor and sample collection

The source of the biological material was a pilot plant applying the anammox process at the RWL Water/Eurotec Water Treatment Technologies, Padova, Italy. The original inoculant was organic waste from a nitrification/denitrification tank of a poultry rendering plant. Samples were collected from the anammox reactor at day 154 and day 189 after inoculation, corresponding, respectively, to a technical performance of 337 and 377 grams of total nitrogen abated per cubic meter per day.

At each time point, 2 ml of sludge were withdrawn and centrifuged for 2 minutes at 10,000 rpm. Pellets were frozen in liquid nitrogen and stored at −80 °C until nucleic acids extraction.

### DNA extraction

The PowerSoil DNA Total Isolation Kit (MOBIO Laboratories Inc. Solana Beach CA, USA) was used for genomic DNA isolation and the manufacturer’s protocol was optimized for the samples as follows: after the bead beating lysis procedure and three cleaning solution steps, TE buffer was added to the C3 solution to its final concentration (10 mM Tris, 1 mM EDTA, pH 8.0) and samples were incubated at 55 °C for 1 hour with proteinase-K (Sigma-Aldrich, 100 ug/ul).

Two Phenol:Chloroform:Isoamyl alcohol (Sigma-Aldrich) and one chloroform purification steps were carried out, followed by an ethanol precipitation with ammonium acetate (2 M final concentration) at −20 °C. Samples were washed twice with 75% ethanol and after drying, pellets resuspended in 100 μl of molecular biology-grade sterile DNAse-RNase free water (Sigma-Aldrich).

Purity and quality of each extracted DNA were measured by NanoDrop 1000 spectrophotometer (Thermo Scientific) and Qubit Fluorometer (Life Technologies) DNA quantification systems. Three extractions were performed for each sample and nucleic acids were finally pooled together.

### 16S rDNA PCR amplification and amplicon sequencing

A universal primer mix for 16S rRNA genes amplification was defined to suit the 454-FLX Titanium sequencing system. A dataset containing over 150,000 16S rRNA gene-related sequences was downloaded from the Ribosomal Database Project (RDP) website, Release-8 (http://rdp.cme.msu.edu/).

Using the *Escherichia coli* 16S rRNA as query, all entries were aligned by nucleotide BLAST and sequences that were not recognized as 16S genes were removed. Any potential pair of primers with a distance between 400 and 600 bases was considered, and pairs amplifying hypervariable regions V3, V4 and V5 were favoured. The primers are a slightly different version of the pair S-D-Bact-0341-b-S-17 and S-D-Bact-0785-a-A-21^51^ which were published after our initial set up.

Three forward primers, degenerated in one position, and three reverse primers, degenerated in three positions, were selected as the most adequate universal oligonucleotides. Their sequences are: S-D-Bact-0343-b-S-15: TACGGGAGGCHGCAG; S-D-Bact-0788-a-A-19: BWGGACTACCVGGGTATCT.

For the 454 amplicon sequencing protocol, sequencing primer-A and multiplex identifier MID were added to the 5′ of the S-D-Bact-0343-b-S-15 mix. Likewise, sequencing primer-B was added to the 5′ of the S-D-Bact-0788-a-A-19 pool.

The a priori virtual performance of the primers (specificity/sensitivity/taxa detection) was first determined *in silico* by two different methods: ProbeMatch software (https://rdp.cme.msu.edu/probematch/search.jsp) and TestPrime (http://www.arb-silva.de/search/testprime/) using the default parameters. Results from both were compared with the similar primers S-D-Bact-0341-b-S-17 and S-D-Bact-0785-a-A-21^52^ and with other primers widely used for 16S-amplicon analysis, U789F-B1046R^53^,^54^ and 515f-806R^55^.

The *in vitro* performance of the custom-synthesized fusion primers (Invitrogen, Life Technologies) was evaluated by PCR and amplicon cloning.

Three replicate PCR-reactions were conducted for each sample. Each reaction was performed in 20 μl using 0.25 U of Phusion High-Fidelity DNA polymerase (Thermo Scientific), the provided Phusion HF Buffer, dNTPs (Promega, 200 μM) and each primer pool (0.3 μM). The reactions were carried out in an Eppendorf Mastercycler EP S set as follows: initial denaturation: 2 minutes at 98 °C, 25 cycles of 20 seconds at 98 °C (denaturation), 45 seconds at 61 °C (annealing) and 15 seconds at 72 °C (extension) followed by 6 minutes at 72 °C as final extension.

PCR products were purified by Agencourt AMPure XP (Beckman Coulter). Samples were measured by NanoDrop, Qubit and Agilent 2100 Bioanalyzer (Agilent Technologies) and the same DNA amount was used for sequencing in a Genome Sequencer Roche 454 GS FLX Titanium.

### RNA extraction and 16S rRNA purification and sequencing

The MOBIO RNA PowerSoil Total RNA isolation kit was used for RNA extraction following the manufacturer’s protocol manual. For each sample, two replicates were performed of the total RNA extraction protocol. Nucleic acids were resuspended in 100 μl of SR7 solution.

The total RNA obtained was run in a 0.8% low-melting point agarose gel (SeaKem LE) at 50 V for approximately 3 hours in a cold room at 4 °C. Slices corresponding to the 16S rRNA band were then cut from the gels. Gel slices were equilibrated with 5 μl TE (10 mM Tris, 1 mM EDTA, pH 8.0) every 1 mg of sample for 20 minutes and washed twice with one volume of TE, 5 minutes for each wash. Three Acid (saturated with 0.1 M citrate buffer pH 4.3 ± 0.2) Phenol:Chloroform:Isoamyl alcohol (Ambion) and one chloroform extraction steps were performed, followed by an overnight ethanol precipitation with sodium acetate (Sigma-Aldrich, 0.3 M) at −20 °C. 16S rRNA samples were washed twice in 75% ethanol and after drying, resuspended in 20 μl of RNAse/DNase-free water (Sigma-Aldrich).

An aliquot of each extracted RNA was used as template for a PCR using 16S universal primers to check for possible excessive proportions of genomic DNA. The replicates of each sample were then pooled together, and purified by RiboMinus (Life Technologies). The protocol was modified as the hybridization step required for the ribo-depletion was bypassed and only the purification/concentration column module was used from the kit. Nucleic acids concentration and purity were measured by NanoDrop, Qubit and by Agilent 2100 Bioanalyzer (Agilent Technologies).

Paired End libraries were prepared following the SOLiD Total RNA seq kit guide and sequenced on a SOLiD 5500xl platform (Life Technologies Inc.). To preserve abundance of the transcripts in the final library, 12 amplification cycles were used after adaptor ligation.

For the electrophoresis, solutions (including water) were prepared by using commercial reagents declared DNase-RNase-free by the manufacturers. Electrophoretic apparati components and slicers were disinfected with 0.01% HCl and RNaseZap (ThermoFisher Scientific).

Samples were checked by Agilent 2100 Bioanalyzer (Agilent Technologies) before and after electrophoresis to ascertain absence of specific signatures of RNA degradation (e.g. differences between 16S-23S rRNAs peaks) and to ensure that purity and quantity were suited to a SOLiD RNA-Seq run.

### Bioinformatics analyses

In terms of reads length, a 454-FLX Titanium produces ~400-bases reads while a SOLiD 5500xl paired-end library yields reads of 70 + 30 bases by an insert of ~100 bases.

454 amplicon reads were processed using Mothur version 1.22.2^19^. Briefly, sequences were filtered for quality score (mean >30), MID and primer sequences were removed and chimeras were checked by Chimera-slayer.

Sequences were analyzed using the rRNA Taxonomy Binning workflow of CAMERA^16^. Annotation was conducted using BLAST and the GreenGenes database as online reference dataset with default settings. SOLiD reads were aligned against the reference dataset (see below) using the PASS software, version 1.64^66^ with the following parameters: identity threshold (-fid): 97, reads length trimming: 35 bases, number of best hits listed: 10. Only pair-end sequences with an estimated distance between 50 and 350 bases were considered as correct.

To correctly assign the SOLiD reads to their taxa, a two-steps procedure was designed. Firstly, only the uniquely-aligned reads, which correspond both to single or paired sequences that present a unique best hit against the reference dataset, were considered to obtain a preliminary group of putative subjects. Only subjects covered for at least 10% of their total length (representing 150 bases of a 1500 full length 16S-rRNA) were selected to build a new 16S rRNA dataset for the next step. In the second step all the SOLiD reads were re-aligned against the newly defined 16S rRNA dataset. Since this dataset represents just a small subset of all the known 16S sequences, many of the initially multi-mapped SOLiD reads presented a unique alignment against the subjects. The final dataset was created selecting those sequences covered for at least 50% of their total length (corresponding to >750 bases in a 1500 bases-long molecule).

As reference dataset, two 16S rRNA-genes databases were downloaded, RDP release 9 and the 16S-rRNA genes sequences from the DDBJ, release 90.1. The RDP database contains both bacterial and archaeal sequences while the DDBJ repository features only bacterial 16S-rRNA genes.

In order to obtain a database that could provide the broadest span of representative biodiversity, the RDP and DDBJ databases were merged together and clustered at decreasing similarity levels of 97.5%, 95%, 92%, 90%, 88%. The cluster analysis was performed using the CD-HIT-EST tool of the CD-HIT package^67^. The reference subjects identified by the SOLiD-reads were classified with the rRNA Taxonomy Binning workflow of CAMERA, using BLAST as aligner and the GreenGenes as online reference database at default settings.

The percentage of reads uniquely-aligned on each subject was considered as an estimator of the bacterial activity in the bioreactor.

A flowchart of the rRNA-seq procedure is shown in Fig. 1.

The project has been deposited in the NCBI database under the code PRJNA274646. Roche-454 reads are available under the codes SRR1791607 and SRR1791615 for day 154 and day 189 sampling times, respectively. SOLiD reads are associated to the codes SRR1791691 and SRX868173, respectively.

### Method accuracy validation by in-silico simulations

To evaluate the data processing and annotation procedure, virtual communities with pre-set taxa identities and abundances (16S-rRNA genes belonging to completely sequenced bacteria) were generated and sequenced *in silico* using the Dwgsim tool (http://sourceforge.net/apps/mediawiki/dnaa/index.php?title=Whole_Genome_Simulation) and tested with the above described pipeline.

To assess a possible coverage-dependent bias (higher reads number for longer genes), the correspondence between extracted subjects and the length of sequences covered by uniquely aligned reads was plotted.

In order to understand if possible biases could be determined by specific features in the analyzed samples, virtual datasets represented by ten randomly-selected subjects from data obtained for day 154 and day 189 samples were produced. A 1000x coverage was imposed for each sequence and their abundances were pre-set as 1/10 of the total. Virtual reads were aligned against the database and subjects were extracted and analyzed using the described strategy applied for the actual anammox bioreactor sequencing data. Comparisons between expected and obtained results were then plotted and inspected.

As regards specific phyla and redundancies related to their over-representation in the reference database, a Proteobacteria validation on five hundred 16S sequences taken from sequenced genomes was carried out. For cases featuring more than one ribosomal operon the one closest to dnaA was considered. Reads were aligned on clustered databases at 95%, 92% and 90% (as previously described) and the relatedness ratio was evaluated considering the expected pre-processed virtual data and the post-alignment results.

The results of all these simulations are shown in Supplementary Material, Note 2.

## Additional Information

**How to cite this article**: Rosselli, R. *et al*. Direct 16S rRNA-seq from bacterial communities: a PCR-independent approach to simultaneously assess microbial diversity and functional activity potential of each taxon. *Sci. Rep.* **6**, 32165; doi: 10.1038/srep32165 (2016).

## Supplementary Material

Supplementary Information

Supplementary Data set S1

## Figures and Tables

**Table 1:** Percentage of sequences obtained by PCR-based analysis and by rRNA-seq at the two sampling times and ratio of the two values pairs (% by PCR/% by rRNA-seq).

Phylum	Day 154 sampling			Day 189 sampling		
	% (by PCR)	% (by RNA)	Ratio by PCR/by RNA	% (by PCR)	% (by RNA)	Ratio by PCR/by RNA
Acidobacteria	1.877	0.084	22.256	3.062	0.053	57.819
Actinobacteria	4.530	0.015	308.903	13.604	0.046	298.464
Bacteroidetes	1.359	0.151	9.020	1.590	0.399	3.989
Armatimonadetes	0.302	5.697	**0.053**	0.412	6.652	**0.062**
Chlorobi	23.598	2.595	9.093	10.984	1.648	6.662
Chloroflexi	2.071	0.867	2.387	1.914	0.978	1.953
Firmicutes	0.669	0.008	81.510	0.383	0.027	14.079
Gemmatimonadetes	4.659	0.206	22.612	6.302	0.177	35.642
Planctomycetes	24.892	87.362	**0.285**	14.694	86.459	**0.170**
Proteobacteria	34.060	2.705	12.593	43.463	3.301	13.166
Candidate Division BRC1	0.388	0.042	9.233	0.736	0.023	32.872
Candidate Division TM7	0.863	0.006	144.614	0.942	0.030	31.799

Only phyla for which a minimum of 10 sequences were available are reported in the table. Ratios resulting in values <1 are highlighted in **boldface**.
