# Supplementary material for: Direct 16S rRNA-seq from bacterial communities: a PCR-independent approach to simultaneously assess microbial diversity and functional activity potential of each taxon
Source: Sci Rep. 2016 Aug 31;6:32165. doi: 10.1038/srep32165 (PMC5006002; doi:10.1038/srep32165)
Supplement: Supplementary Information [file srep32165-s1.pdf]

Direct 16S rRNA seq from bacterial communities: a PCR-independent approach to simultaneously assess microbial diversity and functional activity potential of each taxon.

Riccardo Rosselli[1], Ottavia Romoli[1], Nicola Vitulo[1], Alessandro Vezzi[1], Stefano Campanaro[1], Fabio de Pascale[1], Riccardo Schiavon[1], Maurizio Tiarca[3], Fabio Poletto[3], Giuseppe Concheri[2], Giorgio Valle[1], Andrea Squartini[2].

[1]Department of Biology, University of Padova, Padova, Italy.[2]Department of Agronomy Animals, Food, Natural Resources and Environment, DAFNAE, University of Padova, Legnaro (Padova) Italy.[3]RWL Water/Eurotec WTT, Water Treatment Technologies, S.r.l., Padova, Italy. Correspondence and requests for materials should be addressed to A.S. (email: squart@unipd.it)

*** Correspondence to :** Andrea Squartini, Department of Agronomy Animals, Food, Natural Resources and Environment, DAFNAE, University of Padova, Legnaro (Padova) Italy. squart@unipd.it

Supplementary Note 1 Database clustering threshold effects in the rRNA Sequences alignment and annotation procedure

The described procedure allowed to investigate whether the different similarity thresholds used to cluster the database may affect the reads alignment and in particular the number of uniquely-aligned reads. The clustering procedure decreases the database redundancy as similar sequences tend to be grouped together. This should increase the likelihood that a read could map uniquely.
Interestingly no correlation was found between the number of uniquely-aligned reads and the similarity level used to cluster the databases.

The lowest number of uniquely-aligned reads was obtained for the 97.5% value of similarity threshold for each sample, while the highest number was found at 90% as shown in Tab. 1
The number of the extrapolated records (database subjects as putative target sequences relevant for annotation) appears to be independent from the nearly-tenfold reduction of the database references going to 97.5% to 88% of cluster similarity threshold.

The comparison within each CAMERA taxonomic annotation revealed that a high number of taxa were shared between the different extracted datasets.

The classification resulting for the 95% level of sequence similarity, was the one which gave the highest number of taxonomically-identified database subjects and was therefore chosen as reference to assemble data.

Cluster similarity threshold values	97.5%	95%	92%	90%	88%
Resulting total number of reference subjects in the 16S- database	126,741	62,845	32,455	21,371	14,192
Day 154 aligned reads after subject selection	4,157,738	10,726,911	5,587,652	18,754,964	6,380,678
Day 154 Number of extrapolated subjects	1,347	1,403	1,395	1,242	1,107
Day 189 aligned reads after subject selection	2,226,020	6,809,801	3,139,370	12,004,425	3,507,877
Day 189 Number of extrapolated subjects	1,273	1,319	1,238	1,173	1,017

Table 1. Number of aligned reads against the two pooled databases, clustered at five decreasing levels of similarity from the species rank-level degree (97.5%) downwards.

Supplementary Note 2. Method accuracy validation by in-silico simulations

The validity of the proof of principle hereby presented was tested to exclude possible biases in the reads alignment and subsequent annotation. The aspects considered were the dependency on subject percent coverage and the effect of uneven species richness of the different phyla across the database. For example, the reference 16S sequence database contains a high number of Proteobacteria (encompassing many taxa differing by few nucleotides) and a relative paucity of records for minor phyla. Three different in silico approaches were devised to rule out such possibilities, including the construction of virtual communities of taxa whose number, identity and abundance were pre-set a priori. Tools for virtual sequencing were used to generate reads in the desired format (Dwgsim ; http://sourceforge.net/apps/mediawiki/dnaa/index.php?title=Whole_Genome_Simulation) allowing to compare the expected output with the one resulting from our proposed bioinformatic pipeline. Vitrual datasets of either randomly picked subjects from the same project or featuring 500 proteobacteria with completely sequenced genomes were tested. Results proved the coherence between data annotation and true taxa proportions in the virtually assembled communities.

1) Assessment of possible coverage-related biases

The question behind this check is the following: since the 16S gene has hypervariable regions, could the database redundancy affect the number of reads that align on a subject sequence?
It needs to be recalled that the method is based on the uniquely aligned reads, i.e. a read must map on one and only one subject of the database (although an unlimited number of independent reads could map to the same subject).
In light of that, the issue can be described as follows: when in the database there are two (or more) subjects whose sequences are rather similar (e.g. species of the same genus) a given read could map on both and therefore it would be scored as not-unique and discarded from the count. Vice versa, for a subject which were instead rather characteristic and genetically different from others, there could be a lower chance that a read could map elsewhere. That read will therefore end up being unique and its annotated subject will be kept in the list. Basically in the first scenario a database with high redundancy would offer several highly similar sequences belonging to different subjects which could theoretically enhance the risk for a read to map not-uniquely and be lost. As the redundancy could be higher for species-rich phyla and lower for others, a possible bias could in principle arise.
In order to seize this possibility we performed a specific analysis taking into account the sequence coverage of subjects and the number of reads that aligned on them.
The rationale of the test is the following: if database redundancy affected the scores, when that dataset is clustered at decreasing stringency values of shared homology (from 97% to 90%) its taxa progressively coalesce into higher rank levels (e.g. subjects that at 97% homology are distinguished into different species, get unified into the same genus or even family and orders upon stepping down to 90% similarity cutoffs. Therefore the reads which were not unique at 97% will likely be unique at 90% as a same subject now encompasses sequences that were previously clustered into more different subjects.
This would in turn recruit reads that had been discarded as not-unique when the higher stringency clustering was applied, and therefore more extended portions of the subjects will eventually be covered.
If a redundancy-related bias would exist, one should therefore observe that upon lowering the database clustering stringency (and its consequent redundancy) an higher number of reads should map on longer and longer portions of the subjects. To verify if this could apply we checked, the possible correlation between the length of the subjects' sequences that were covered by the reads and the number of reads that aligned uniquely on them. The result is shown in Fig. 1.

As the graphs show there is no hint of correlation between number of reads and subject coverage and no pattern changes upon lowering the database clustering stringency. Therefore neither redundancy nor database similarity aspects appear to affect the data analysis procedure.

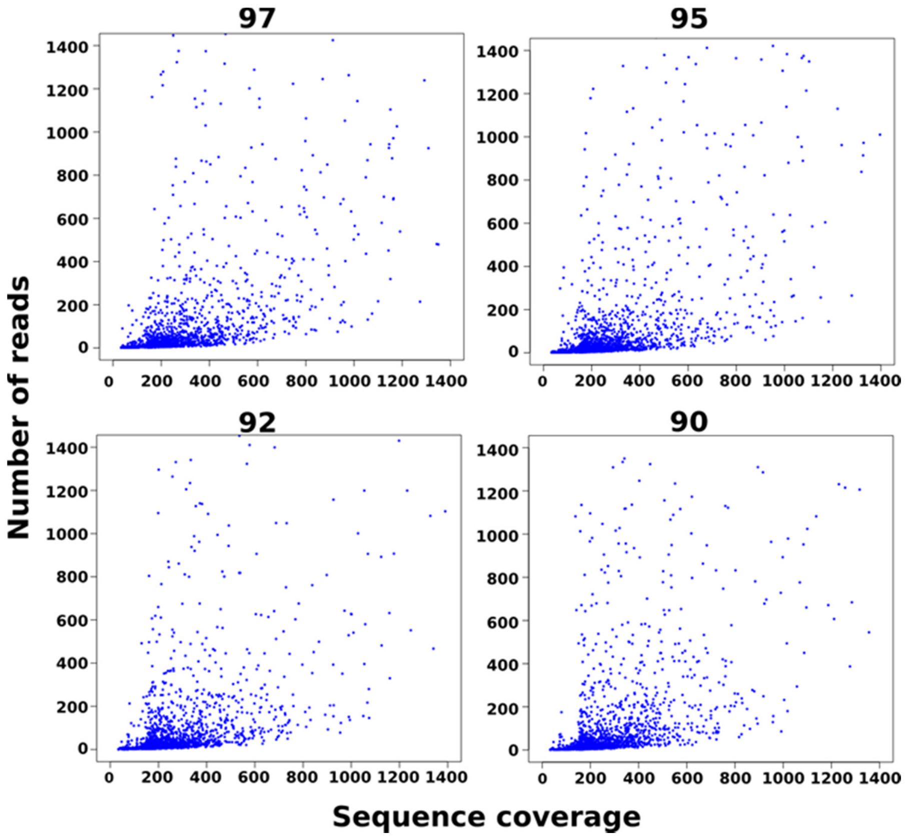

Fig. 1 Plotted correspondence between extracted database subjects and the respective coverage (length of sequence covered by uniquely aligned reads). Data refer to the first time point and show the result at four decreasing levels of database clustering (from top: 97.5, 95, 92 and 90% respectively)

As can be seen, no line can be fitted across the points, i.e. there is no correlation between the number of mapped SOLiD reads and the length of the covered region. Most sequences are encompassed between 200 and 400 bases and no relationships link the number of reads to the covered length within the subjects.

2) Tests with virtual communities of known composition

In order to verify that the SOLiD-based analysis carried out in this work from the anammox bioreactors were respectful of their true quantitative proportions, the whole procedure was run on series of in-silico assembled datasets obtained upon simulating a SOLiD sequencing with a priori known coverage and number of species. As both taxonomical identity and members proportions were pre-set, this allowed to seize the accuracy of the proposed protocol.

A whole virtual SOLiD-paired-end sequencing was performed with Dwgsim (http://sourceforge.net/apps/mediawiki/dnaa/index.php?title=Whole_Genome_Simulation).

Two pairs of virtual datasets represented by ten randomly-selected subjects from the final data obtained for day 154 and day 189 samples were produced. A 1000x coverage was imposed for each sequence and the abundance of each in the virtual dataset was pre-set as 1/10 of the total.

Virtual sequences were aligned against the database and subjects were extracted and analyzed using the same strategy applied for the real anammox bioreactor sequencing data. As the ten subjects were selected randomly some occurred within the same taxonomic group (i.e. the resulting number of annotated taxa could be ≤10). As the subjects had different lengths, being the desired coverage imposed to 1000x the resulting number of reads matching on each subject varies accordingly. The number of reads was normalized on their length and the data expressed in percentage. When more than one sequence belonging to the same taxonomic level was present, the percentage shown was evaluated considering all the related virtual sequences. The outputs were reported graphically showing the percent number of reads encompassing all subjects that share that given taxonomical classification. Where in the database a denomination of a given taxon were not yet present at the identity threshold chosen (i.e. for yet to describe species) the operational technical unit (OTU) output applied.

The results of these comparisons are presented in Fig.2 showing at different taxonomical hierarchical level, the percentage of reads obtained by performing the alignment and annotation protocol (green bars) side by side with the corresponding expected number (blue bars). Values are expressed in percentage of the total number of reads considered. In essence the accuracy of the approach can be visualized by comparing the values of the green histograms with their adjacent blue counterparts.

As the figure shows, highly coherent and faithful representation of the known true proportion was obtained for the majority of cases, endorsing the validation of the approach and ruling out biases towards given phyla.

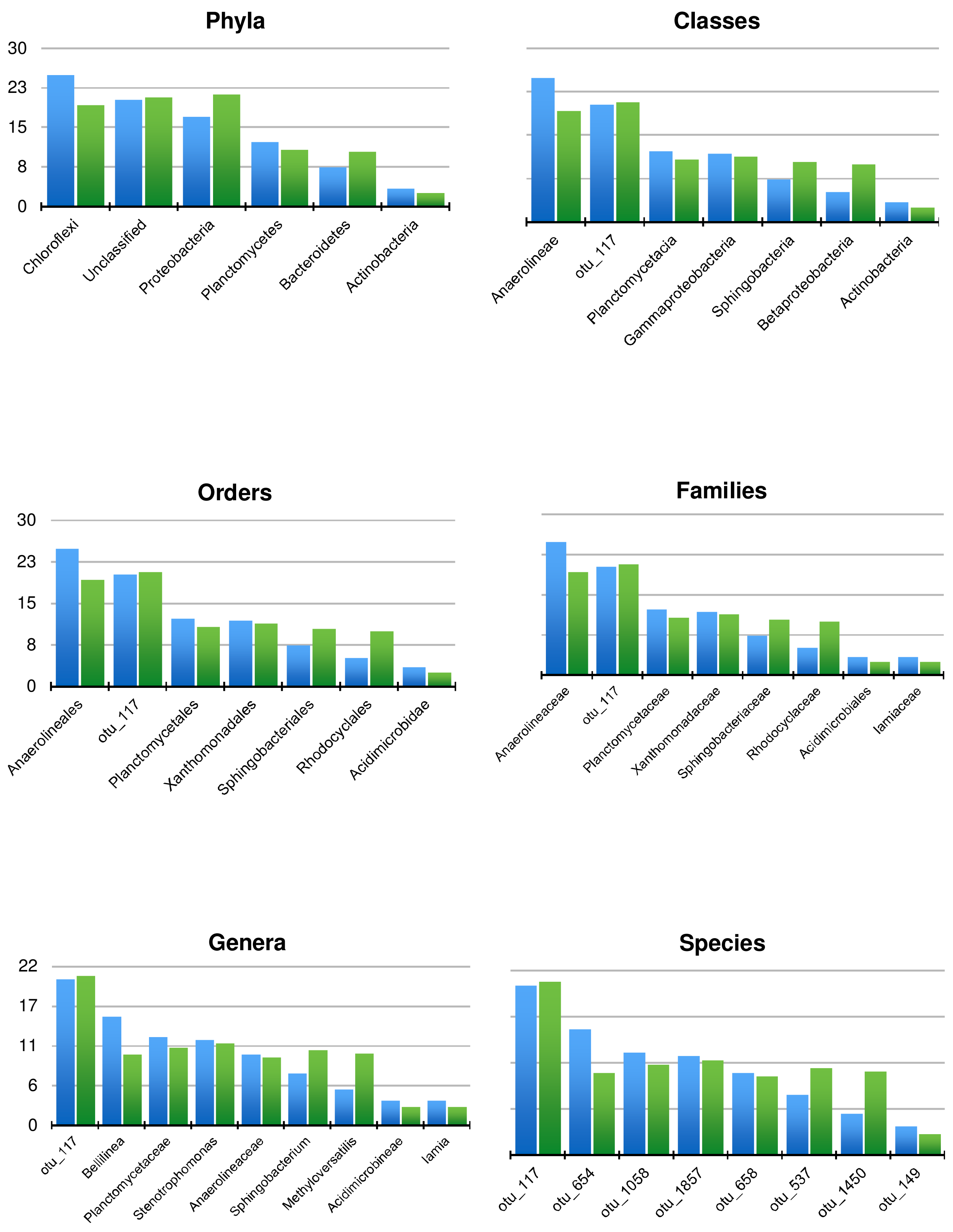

Fig. 2. Comparison between expected (blue) and obtained (green) annotation results upon running the analysis on the virtual sequencing dataset of the random sequences at day 154. Percent of reads pointing to given taxonomical annotations at different systematic hierarchy levels are reported. The same procedure carried out with the dataset from day 189 gave essentially the same results (not shown).

3) Proteobacteria-specific validation on 500 16S sequences taken from sequenced genomes

A further independent check of the method was done with the Proteobacteria since this phylum was the most divergently-represented group in the comparison between the PCR-based and the direct rRNA seq approaches. Moreover, due to its implication in several fields (from laboratory to clinical to environmental), Proteobacteria-related ribosomal sequences are highly represented in the databases with several similar sequences belonging to the same species.
In order to understand if a possible bias affecting uniquely- and not-uniquely aligned reads could be related to the particular taxon of Proteobacteria, a further analysis was performed.
A group of five hundred 16S-rRNA gene sequences was selected within available completely-sequenced proteobacterial chromosomes. Where more than one ribosomal operon was present, the one closest to *dnaA* was considered for the analysis. Virtual sequencing and data analysis were carried out as previously described. Clustered databases at 95%, 92% and 90% of similarity were considered for the alignment and the original sequences header in the databases was used for the subjects-classification process. A related ratio was evaluated considering the expected pre-processed virtual data and the post-alignment results. Results are shown in Fig. 3

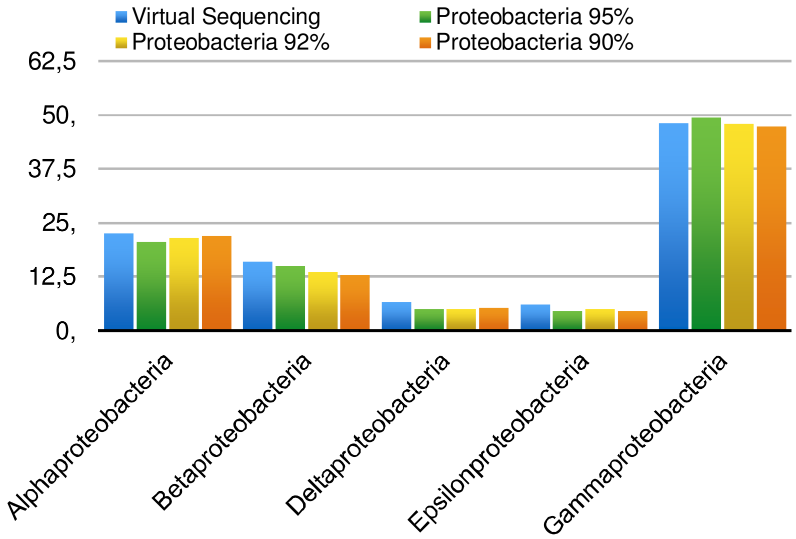

Fig. 3. percent of reads yielding the classification within five classes of the phylum Proteobacteria upon the virtual sequencing of 16S gene sequences from 500 available genomes. The expected (a priori known) value is shown In blue and those resulting from the annotation routine proposed in this work on the reads generated by the simulated sequencing are plotted sideways. Results obtained at the three levels of clustering of the database (95, 92 and 90%) are shown.

As regards the lower taxonomic levels their graphical representation would be impractical dealing with 500 taxa, but the result was in line with the other analyses shown here and backed up by a robust statistical solidity with E values spanning from $2.2x10^{-16}$ to $1.8x10^{-16}$.
